# Comparison of cardiovascular risk factors between sri lankans living in kandy and oslo

**DOI:** 10.1186/1471-2458-10-654

**Published:** 2010-10-29

**Authors:** Sampath UB Tennakoon, Bernadette N Kumar, Danasela B Nugegoda, Haakon E Meyer

**Affiliations:** 1 Department of General Practice and Community Medicine, University of Oslo, Oslo, Norway; 2 Department of Community Medicine, University of Peradeniya, Peradeniya, Sri Lanka; 3 Norwegian Institute of Public Health, Oslo, Norway

## Abstract

**Background:**

South Asians living in western countries are known to have unfavourable cardiovascular risk profiles. Studies indicate migrants are worse off when compared to those living in country of origin. The purpose of this study was to compare selected cardiovascular risk factors between migrant Sri Lankans living in Oslo, Norway and Urban dwellers from Kandy, Sri Lanka.

**Methods:**

Data on non fasting serum lipids, blood pressure, anthropometrics and socio demographics of Sri Lankan Tamils from two almost similar population based cross sectional studies in Oslo, Norway between 2000 and 2002 (1145 participants) and Kandy, Sri Lanka in 2005 (233 participants) were compared. Combined data were analyzed using linear regression analyses.

**Results:**

Men and women in Oslo had higher HDL cholesterol. Men and women from Kandy had higher Total/HDL cholesterol ratios. Mean waist circumference and body mass index was higher in Oslo. Smoking among men was low (19.2% Oslo, 13.1% Kandy, P = 0.16). None of the women smoked. Mean systolic and diastolic blood pressure was significantly higher in Kandy than in Oslo.

**Conclusions:**

Our comparison showed unexpected differences in risk factors between Sri Lankan migrants living in Oslo and those living in Kandy Sri Lanka. Sri Lankans in Oslo had favorable lipid profiles and blood pressure levels despite being more obese.

## Background

Cardiovascular disease (CVD) risk profile of South Asians living in western countries is characterized by low High Density Lipoprotein (HDL) cholesterol, central obesity and increased diabetes mellitus together with higher rates of myocardial infarctions, re-infarctions and higher mortality rates from Coronary Heart Disease (CHD) [1-4]. By grouping South Asians together, some studies may have overlooked inherent differences amongst them [2].

At present South Asia is experiencing a rapid increase in CVD particularly in the urban areas and among higher socioeconomic classes [5-10]. Studies comparing migrant Indians in UK and USA with those living in India observe migrants having higher mean total cholesterol, triglycerides and Body Mass Index (BMI) but no consistent difference in HDL [11,12].

In Sri Lanka coronary heart disease (CHD) is a main cause of morbidity and mortality [13,14]. Sri Lankan studies suggest concentration of risk factors in urban areas and higher socioeconomic classes with an increasing prevalence among younger people [8-10,15]. A diet rich in carbohydrates and saturated fats (coconut is the major supplier of fat energy) but low in protein may contribute to the worsening burden of CVD and diabetes [9,16]. It has been previously reported from Oslo, Norway that Sri Lankan migrants have lower HDL cholesterol and higher triglycerides compared to Vietnamese, Iranians and ethnic Norwegians[17]. The prevalence of central obesity was highest among Sri Lankan and Pakistani women in Oslo and both men and women had higher Waist to Hip ratios for any given BMI compared to other immigrant groups [18]. To our knowledge, no studies comparing Sri Lankan migrants and a native group in Sri Lanka have been published. Our study compares cardiovascular risk factors from a population based study in Kandy Sri Lanka with data from Sri Lankans participating in the Oslo Immigrant Health Study. The study design and implementation in Kandy was as similar as possible to the Oslo study to facilitate the comparison.

## Methods

### Study population - Oslo, Norway

The population based, cross sectional Oslo health study (HUBRO) and Oslo immigrant health study were conducted between 2000 and 2002 by the Norwegian Institute of Public Health and the University of Oslo [17]. Both studies used the same protocol. In HUBRO, all Oslo residents born in 1924, 1925, 1940, 1941, 1955, 1960 and 1970 were invited. In the Oslo immigrant health study, all those born between 1942 and 1971in Sri Lanka, Turkey, Iran Vietnam and a 30% random sample of Pakistanis living in Oslo were invited, except for those who previously had been invited to HUBRO [19]. An invitation and the main questionnaire were sent to participants 2 weeks before the screening followed by a reminder to non responders. In both studies the questionnaires were also available in the appropriate languages of the five immigrant groups. Here we have included participants from both studies born in Sri Lanka between 1940 and 1971, and in this group the response rate was 50% in HUBRO (143 participants) and 50.9% in the Oslo immigrant health study (1002 participants) [19]. The majority of the Sri Lankans (99%) in Oslo belonged to the Tamil ethnic group.

### Study population - Kandy, Sri Lanka

The population based cross sectional study in Kandy was conducted in the municipal council area between August and December 2005 among ethnic Tamils. The target was 300 men and women between the ages of 30 and 60 years. The government electoral list for 2004 in which those above 18 years are required to register was the sampling frame [8-10,20]. Tamils were identified by their family names and selected through simple random sampling. All the selected persons were then invited at house visits after verification of ethnicity and age. Of those invited, 74 percent of the men and 92 percent of the women participated.

### Data collection

Data collection in Kandy followed the Oslo study with a very similar protocol. In Oslo, participants completed a questionnaire, with or without assistance, while participants in Kandy were interviewed using a structured questionnaire. In both studies years of education, personal history of chronic diseases and medication and smoking habits were recorded. The Norwegian population register provided information on age and gender and country of birth which was taken as the county of origin [19]. In Kandy date of birth was recorded at the interview while gender was provided by the electoral list. Body weight and height were measured with electronic Height and Weight Scale in Oslo and a Salter medical scale and a Statometer in Kandy, with the participants wearing light clothing without shoes. BMI (kg/m^2^) was calculated accordingly [19]. Waist circumference, at the midpoint between the iliac crest and lower margin of ribs was measured with the subject standing and breathing normally to the nearest 0.1 cm with the same steel measuring tape utilised in both studies.

Systolic and diastolic blood pressures were measured three times at one-minute intervals in mmHg by an automatic device (DINAMAP, Criticon, Tampa, USA) in Oslo and with a mercury sphygmomanometer in Kandy. The mean of the last two recordings were used in this paper. Hypertension was defined as systolic blood pressure ≥ 140 mmHg or diastolic blood pressure ≥ 90 mmHg or being on blood pressure lowering drugs.

Non-fasting blood samples were collected and serum total cholesterol, serum HDL cholesterol and serum triglycerides were measured directly by an enzymatic method. This was done at the Department of Clinical Chemistry, Ullevål University Hospital, Oslo, Norway which was the reference laboratory, (Hitachi 917 auto analyzer, Roche Diagnostic, Switzerland) and ESPEE laboratory Kandy Sri Lanka (COBAS MIRA 36-3122 auto analyzer).

### Cross calibration of serum analysis

For purposes of comparison, serum from a random sample of 14 persons from the Kandy study was re-analyzed at the reference laboratory in Oslo.

As the Kandy results for total cholesterol and HDL cholesterol showed systematic differences from the Oslo results a further 182 samples were re-analyzed at the reference laboratory, including 8 of the initial 14. Adjustments in total cholesterol and HDL cholesterol values from the Kandy study were thus made according to the reference laboratory scale. Triglyceride values did not differ between the two laboratories.

### Ethical considerations

The Higher Degrees and Research Ethics committee of the University of Peradeniya, Sri Lanka approved the Kandy study. HUBRO and the Oslo Immigrant Health Study were approved by the Norwegian Data Inspectorate and cleared by the Regional Committee for Medical Research Ethics.

### Data analysis

Combined data were analyzed by SPSS version 16 using linear regression and UNIANOVA methods with all variables adjusted for age, except age. Triglycerides were also adjusted for time since last meal. Regression analyses assumptions (linearity and similar variance over different levels of the dependent variable) were checked by inspecting plots of residual against predicted values.

## Results

A total of 685 men and 460 women from Oslo and 103 men and 130 women from Kandy were included in the analysis whose general characteristics are described in Table 1.

**Table 1:** Characteristics of the study populations in Oslo, Norway and Kandy, Sri Lanka (Age adjusted means and prevalences*)

	Oslo	Kandy	P**
**MEN**			
N	685	103	
Age (years)	40.0	46.4	< 0.01
Education (years)	13	10	< 0.01
Total cholesterol (mmol/l)	5.4	5.2	0.18
HDL cholesterol (mmol/l)	1.07	0.89	< 0.01
Total/HDL cholesterol ratio	5.3	6.3	< 0.01
Triglyceride (mmol/l)***	2.6	2.6	0.95
Height (cm)	168	163	< 0.01
Body Mass Index (kg/m^2^)	25.7	22.5	< 0.01
Waist circumference (cm)	89	81	< 0.01
Systolic blood pressure (mmHg)	126	129	< 0.02
Diastolic blood pressure (mmHg)	77	83	< 0.01
Current smoking (%)	19	13	0.16
			
**WOMEN**			
N	460	130	
Age (years)	39	45.6	< 0.01
Education (years)	12	10	< 0.01
Total cholesterol (mmol/l)	5.0	5. 3	< 0.01
HDL cholesterol (mmol/l)	1.21	0.98	< 0.01
Total/HDL cholesterol ratio	4.3	5.7	< 0.01
Triglyceride (mmol/l)***	1.8	2.2	< 0.01
Height (cm)	155	150	< 0.01
Body Mass Index (kg/m^2^)	26.8	24.7	< 0.01
Waist circumference (cm)	84	80	< 0.01
Systolic blood pressure (mmHg)	119	129	< 0.01
Diastolic blood pressure (mmHg)	69	82	< 0.01
Current smoking (%)	0	0	

*The model is evaluated at mean age of 40.7, P** = significance test for equality, ***triglycerides also adjusted for time since last meal

Compared to Oslo, mean age was higher and mean years of education lower in Kandy (Table 1).

Men in Oslo had higher mean HDL cholesterol compared to men in Kandy. Their mean total to HDL cholesterol ratio was lower whereas total cholesterol and triglycerides were similar to Kandy. Prevalence of unfavourable HDL was higher among Kandy men while prevalences of high total cholesterol, total to HDL cholesterol ratio and triglycerides were similar (Table 2). Oslo women too had higher mean HDL cholesterol and lower total cholesterol, total to HDL cholesterol ratios and triglycerides. Prevalence of unfavourable blood lipids was higher in Kandy women.

**Table 2:** Prevalence (%) of selected risk factors among men and women from Oslo and Kandy (Age adjusted)

	Oslo Prevalence	Kandy Prevalence	P*
**Men**			
N	685	103	
High Total cholesterol ≥ 6.2 mmol/l	19.2	20.0	0.89
Low HDL ≤ 0.9 mmol/l	27.8	58.3	< 0.01
High Total to HDL cholesterol ratio ≥ 4.4	70.1	77.9	0.39
High Triglyceride ≥ 2.7 mmol/l**	33.1	39.7	0.24
General obesity ≥ 25 kg/m^2^	58.3	19.6	< 0.01
High Waist circumference ≥ 90 cm	43.7	16.2	< 0.01
Hypertension- SBP ≥ 140 mmHg, DBP ≥ 90 mmHg or on antihypertensive	17.3	33.3	< 0.01
			
**Women**			
N	460	130	
High Total cholesterol ≥ 6.2 mmol/l	8.9	25.9	< 0.01
Low HDL ≤ 1.0 mmol/l	24.7	53.3	< 0.01
High Total to HDL cholesterol ratio ≥ 4.4	43.0	69.0	< 0.01
High Triglyceride ≥ 2.2 mmol/l**	25.7	35.8	< 0.01
General obesity ≥ 25 kg/m^2^	68.2	48.2	< 0.01
High Waist circumference ≥ 80 cm	66.0	46.2	< 0.01
Hypertension- SBP ≥ 140 mmHg, DBP ≥ 90 mmHg or on antihypertensive	9.3	38.2	< 0.01

The model is evaluated at mean age of 40.7, P* = significance test for equality

**triglycerides adjusted for time since last meal,

SBP = systolic blood pressure. DBP = diastolic blood pressure

Men and women in Oslo were about 5 cm taller than their counterparts in Kandy. Mean Body Mass Index was higher in Oslo by about 2 and 3 units respectively among women and men. The Oslo sample also had larger mean waist circumferences.

No women smoked and in men 19% in Oslo and 13% in Kandy reported current smoking (p = 0.16).

Mean systolic and diastolic blood pressure and prevalence of hypertension was higher in Kandy. Current use of antihypertensive medications was reported by 9% of men and 11% of women in Oslo and 12% of men and 17% of women in Kandy.

Triglycerides increased by years of education among men in Kandy. No other statistically significant relations between education and blood lipids in men were found (Table 3). Among Kandy women, mean HDL increased with years of education while in Oslo a decrease in mean total to HDL cholesterol ratio and an increase in mean HDL were suggested.

**Table 3:** Selected risk factor associations with years of education in Kandy and in Oslo (age adjusted)

		Men				Women			
			
Education (years)		0-8	9-12	> 13	p*	0-8	9-12	> 13	p*
									
Participants (Number)	Oslo	29	330	295		35	225	126	
	Kandy	22	62	19		35	71	24	
									
HDL cholesterol (mmol/l)	Oslo	1.01	1.08	1.07	0.81	1.20	1.19	1.25	0.06
	Kandy	0.91	0.91	0.90	0.75	0.96	0.96	1.05	0.05
									
Total cholesterol to HDL ratio	Oslo	5.8	5.3	5.3	0.52	4.5	4.3	4.2	0.09
	Kandy	6.3	6.3	6.9	0.57	6.1	6.0	5.3	0.32
									
Triglycerides** (mmol/l)	Oslo	2.9	2.6	2.5	0.84	1.9	1.7	1.7	0.78
	Kandy	1.8	2.7	3.0	< 0.03	2.1	2.2	2.3	0.74
									
Waist circumference (cm)	Oslo	92.6	88.5	88.7	0.13	83.6	84.5	82.8	0.13
	Kandy	75.4	81.9	88.2	< 0.01	78.4	82.5	81.3	0.72
									
BMI (kg/m^2^)	Oslo	26.7	25.7	25.8	0.85	27.3	27.2	26.5	0.16
	Kandy	20.9	23.1	23.7	< 0.01	23.7	26.3	24.7	0.80
									
Height (cm)	Oslo	167.6	167.3	168.2	0.22	154.9	155.2	156.2	< 0.01
	Kandy	160.3	162.5	164.5	< 0.01	147.0	150.0	150.5	< 0.03
									
Systolic blood pressure (mmHg)	Oslo	124.4	126.5	126.5	0.95	119.1	119.4	115.8	< 0.01
	Kandy	123.2	128.5	133.0	< 0.01	123.6	131.1	132.6	< 0.04

The model is evaluated at mean age of 40.7, p* = significance test for trend, **triglycerides also adjusted for time since last meal

BMI and waist circumference increased with years of education among Kandy men but not women. In Oslo there was no clear association between education and waist circumference or BMI, except that the men with the least education had higher waist circumferences. Height increased with education in all groups except for men in Oslo.

Systolic blood pressure showed a significant increase with education in both men and women from Kandy. Men from Kandy and Oslo with the least education had similar levels of blood pressure while the gap widened at the other end. Women too had a somewhat similar pattern. Among Oslo women, those with the highest education had lowest systolic blood pressure.

Smoking was not clearly associated with education although those with the highest level of education in Kandy had the lowest prevalence of 1.3% (P (equality) = 0.07, data not shown).

## Discussion

The Kandy sample had less favourable lipid profiles compared to Oslo with lower HDL cholesterol and higher total to HDL cholesterol ratios. Kandy women also had higher triglycerides. Parameters of elevated blood pressure were significantly higher in Kandy. On the other hand the Oslo sample was heavier and had larger waist circumferences. In Kandy those with more years of education appeared to be worse off with regard to blood pressure, than those with lower years of education. Among Kandy men obesity and triglycerides were positively related to education. Smoking was low among men and no women reported smoking.

In our study the Oslo migrants had a better blood lipid profile than their counterparts in Kandy. Given the higher obesity indices, unfavourable lipid profiles would have been expected among the migrants [21]. A possible increase in protein intake and changes in the type of fat could attribute for the favourable lipids among the migrants [22,23]. Ethnic Norwegian men showed lower triglyceride levels and tended to have higher HDL compared to immigrants from Sri Lanka in Oslo despite a higher BMI [17]. It has also been observed that despite increasing body weight the CVD burden has decreased in Norway, while blood lipids and the quality of the diet has improved over the last 30 to 40 years [24]. Sri Lankan migrants to Oslo might be consuming a diet relatively rich in fatty fish and unsaturated fats contributing to the improved lipid profiles. Compared to a previous study among males in Kandy the present study observes similar mean HDL cholesterol and total cholesterol in Kandy [8].

Few studies compare South Asian migrants from Western Countries with those in the country of origin. A study comparing Gujarat migrants in the UK with Gujarat's in India from a similar geographic, cultural and genetic background found higher serum total cholesterol, triglycerides, general and central obesity and blood pressure among the migrants [11]. On the other hand, migrants had higher HDL and smoked less.

In the Gujarat study, shorter stature, lower BMI and lower prevalence of overweight and central obesity was found among the non-migrant group, similar to our study [11]. Higher prevalence of overweight among migrants may be the result of increased caloric intake among them following migration. The prevalence of overweight among the migrants in our study was similar to the value found among migrant south Asians to the UK [25]. A greater proportion of women were overweight compared to men in both Kandy and Oslo which is consistent with other studies among Sri Lankans in Sri Lanka [26]. Compared to other studies in Sri Lanka, men in Kandy had a similar prevalence of obesity whereas women had higher abdominal obesity but similar mean BMI's [10,20,27]. A recent study among the general population of Sri Lanka reports lower mean BMI and lower prevalence of overweight and obesity than found in our study for both men and women but the same study reports higher obesity indices for urban areas [26]. An increase in height by education has been observed among immigrants in UK as shown in our study in Kandy [25]. Stature is an indicator of childhood availability of nutrition and may be an indicator of parental socio-economic status [25,28].

The migrants in our study had lower blood pressures in contrast to the Gujarati Study where the migrants had higher blood pressure [11]. Higher blood pressure in Kandy can probably not be explained by lower detection since a higher proportion was on antihypertensive medication and health care is provided free of charge to the patient in Sri Lanka. Kandy participants in our study had higher mean systolic blood pressure compared to other studies in Sri Lanka [8-10]. It is interesting to note that systolic blood pressure of males in the lowest and middle education categories were similar between Oslo and Kandy while higher educated people in Kandy had much higher systolic blood pressure compared to their counterparts in Oslo. This finding is compatible with the finding of higher CVD risk among upper socio-economic groups in developing countries [7].

Compared to the Gujarat study where more natives were current smokers no significant difference was observed between Oslo and Kandy. In Oslo, smoking prevalence among Sri Lankan men was lower than ethnic Norwegians which corresponds to studies from UK where South Asian migrants were not smoking as much as the ethnic British [2,4,17]. A study from southern Sri Lanka in 2005 found similar smoking rates as seen here in Kandy but a much higher prevalence was found in Kandy in 1995 [10,29]. A recent study by Katulanda et al reported much higher prevalence of smoking among men in the general population of Sri Lanka than found in our study [15]. All women were non-smokers consistent with low smoking prevalence among south Asian women in UK, India and Sri Lanka [2,11,29].

In Kandy, men with more years of education were worse off with regard to triglycerides, obesity indices and systolic blood pressure. Similar observations have been made in other developing countries where higher socio-economic standards were associated with unfavourable CVD risk factors [7,30].

### Strengths and weaknesses

By design the two studies are similar. Data collection in Kandy was carried out 3-5 years after Oslo. In Kandy no program to change CVD risk factors in the community took place and no major economical or social conditions change occurred during this time period. Therefore these factors may not have implications on the results of the study.

An important objective of our study is the comparison of two groups with similar ethnic and cultural backgrounds. On the other hand the Tamils in Oslo are not necessarily representative of all Sri Lankan Tamils. Migrants are in general a selected group of people who are healthier and also in most cases socio-economically better off. This is demonstrated in our study by the higher level of education and higher stature among the immigrants which could indicate better socio-economic standards during childhood giving them an advantage socially and economically by being the fittest in the community [25].

The significant difference in mean age of the two groups may not have contributed to the significant differences since an analysis of lipids and blood pressure of the groups divided at median age revealed no consistent pattern of the older group having higher rates of the risk factors.

Despite biochemical measurements being done at two different laboratories, a cross calibration was done to enable a valid comparison.

The blood pressure data should be interpreted with caution as blood pressure measurement techniques differed between the studies although similar conditions; non-fasting and resting prevailed in both places, The Oslo study used the automatic Dinamap method which is known to measure a lower diastolic blood pressure [31]. However, the large differences in systolic blood pressure between Kandy and Oslo can probably not be accounted for by the measurement methods alone, especially since the systolic blood pressure among the lower educated in Kandy and Oslo was similar.

The lower rate of participation among Oslo group is an issue of concern as one of the factors affecting attendance in Oslo was level of education http://www.fhi.no/artikler/?id=53584. Therefore we cannot exclude selection bias. However, no significant gradients between education and risk factors in Oslo were observed, except for height and systolic blood pressure in women. An analysis of the effects of non-participation in HUBRO and the Oslo Immigrant Health Study concluded that prevalence estimates might be valid despite considerable nonattendance [32].

The electoral list, used for random sample generation in Sri Lanka in earlier studies, provided the sample frame [8,9]. Simple random sampling maximized the representation of the population studied. Participation was high at 92.2% for women and 74.1% for men which limits the selection bias. On the other hand a larger sample would have increased the power to detect smaller differences in risk factors, and the small sample size in Kandy may have masked some differences between the two groups. No data on non-participants was collected which is a shortcoming. Using surnames to identify Tamils in Kandy has limitations.

## Conclusions

Compared to Kandy, we found migrant Sri Lankans in Oslo to have higher rates of general and central obesity, which might be due to life-style changes following migration [11]. Higher HDL and lower total to HDL cholesterol ratios in the Oslo group could also be attributed to life style changes. Lower HDL and higher total cholesterol to HDL ratios among Kandy men and women and also higher total cholesterol among women put them at a higher risk for cardiovascular disease in spite of lower BMI and lower waist circumferences. Higher triglycerides in Kandy, despite of lower BMI, in contrast to other studies comparing migrants and those in country of origin, is noteworthy since a triglycerides are known to be positively associated with BMI [11]. Men in Kandy with more education seem to be at a higher risk than those with lower education by way of higher triglycerides, obesity and blood pressure, consistent with other studies [7,26,30]. Our study shows that management of obesity among Sri Lankan migrants needs immediate attention in Oslo while there is a great need for management of unfavourable serum lipids in Kandy.

## Competing interests

The authors declare that they have no competing interests.

## Authors' contributions

SUBT participated in the design of and collection of data in the Kandy study and the statistical analysis and drafting of the manuscript. HEM conceived of the study and participated in the design and coordination of the studies in Oslo and Kandy and in statistical analysis and drafting of the manuscript. BNK participated in the design and coordination of the study and drafting of the manuscript. DBN participated in the coordination of the Kandy study and drafting of the manuscript. All authors read and approved the final manuscript.

## Pre-publication history

The pre-publication history for this paper can be accessed here:

http://www.biomedcentral.com/1471-2458/10/654/prepub
